# A Unified Method for Detecting Phylogenetic Signals in Continuous, Discrete, and Multiple Trait Combinations

**DOI:** 10.1002/ece3.71106

**Published:** 2025-03-20

**Authors:** Liang Yao, Ye Yuan

**Affiliations:** ^1^ Collaborative Innovation Center of Recovery and Reconstruction of Degraded Ecosystem in Wanjiang Basin Co‐Founded by Anhui Province and Ministry of Education Anhui Normal University Wuhu China; ^2^ Provincial Key Laboratory of Biotic Environment and Ecological Safety in Anhui School of Ecology and Environment, Anhui Normal University Wuhu China

**Keywords:** Brownian motion model, continuous variable, discrete variable, Markov model, multiple trait combination, phylogenetic signal

## Abstract

Phylogenetic signals are widely used in ecological and evolutionary studies. Trait data used to detect phylogenetic signals can be continuous or discrete, but existing indices are designed for either type, not both. Moreover, most existing methods can only perform phylogenetic detection of individual traits, despite the fact that biological functions are often the result of interactions among multiple traits. Some attempts to detect phylogenetic signals across multiple trait combinations have employed alternative indicators, which may not align perfectly with the rigorous criteria for defining phylogenetic signals. In this study, we developed a new index (the *M* statistic) to detect phylogenetic signals for continuous traits, discrete traits, and multiple trait combinations. This capability is inherited from Gower's distance, which is used in the calculation of the *M* statistic to convert various types of traits into distances. The *M* statistic strictly adheres to the definition of phylogenetic signals and detects them by comparing these distances from phylogenies and traits. Using simulated data, we compared the performance of our new approach with that of existing commonly used indices. The results show that our method is not inferior to the existing methods. It performs well in handling continuous variables, discrete variables, and multiple trait combinations. We used trait data of turtles (Testudines) to demonstrate the utility of our new method. We suggest this new index as an original method for the detection of phylogenetic signals across various variable types. We provide an R package called “phylosignalDB” to facilitate all calculations.

## Graphical abstract

We developed a method to detect the phylogenetic signals in continuous, discrete, and combinations of multiple traits. The new method strictly adheres to the definition of the phylogenetic signals and detects them by comparing distances from phylogenies and traits. We provide an R package called “phylosignalDB” to facilitate all calculations.

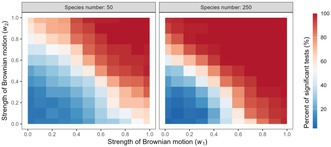

## 1 Introduction

Ecological and evolutionary studies have shown that closely related species tend to have more similar trait values than distantly related species (Burns and Strauss 2011). These observations demonstrate that the genetic relationship or evolutionary history (phylogeny) of the species has a significant effect on their traits. All species inherit and retain some traits from their historical ancestors, resulting in similar traits among species of common ancestry. In statistical terms, this phenomenon is known as statistical non‐independence or statistical dependence (Harmon 2019). The term phylogenetic signal was proposed to measure and test this phylogenetic dependence and is defined as the “tendency for related species to resemble each other more than they resemble species drawn at random from the tree” (Blomberg and Garland Jr 2002). To measure and test phylogenetic signals, ecologists and evolutionists have attempted to find solutions in other disciplines. They found that the non‐independence of species is analogous to spatial autocorrelation in geography and spatial statistics, a concept that was later adapted for measuring and testing phylogenetic signals and resulted in the development of metrics such as Abouheif's *C*_mean_ (Abouheif 1999) and Moran's *I* (Nabout et al. 2010). In addition, models of biological evolution and many simulation tools have been developed. An alternative approach is to employ a specific model of evolution as a null or reference model, typically a Brownian motion model for trait evolution. Metrics, such as Pagel's *λ* (Pagel 1999) and Blomberg's *K* (Blomberg et al. 2003), are constructed to measure the fit between the observed trait values and the theoretical distribution determined by the evolutionary model. Calculations of these indices have been included in popular R packages for ecology and evolution, such as phylosignal (Keck et al. 2016), picante (Kembel et al. 2010), ape (Paradis and Schliep 2019), and phytools (Revell 2024), and are widely used in the comparative analysis of traits among species.

Trait data used to detect phylogenetic signals can be continuous (e.g., weight of an animal, leaf area of a plant) or discrete (e.g., color of the petals of a plant, nesting activity of an animal). However, most existing methods detect phylogenetic signals only for continuous traits (Münkemüller et al. 2012) and cannot be directly applied to discrete traits. A few methods have been developed specifically for discrete traits, such as the *D* statistic (Fritz and Purvis 2010) and *δ* statistic (Borges et al. 2019). The *D* statistic is only applicable to binary traits. Furthermore, the binary traits analyzed must have evolved according to the Brownian motion threshold model (Harmon 2019). The *δ* statistic, which is based on Shannon entropy, is theoretically applicable to any discrete trait without specific requirements for the number of states or the evolutionary pattern of the trait. However, both indices are specifically tailored for discrete traits and are not suitable for continuous traits. Notably, using different methods based on distinct principles to simultaneously test for phylogenetic signals in a range of traits hinders the comparability of the results to some extent. This underscores the need to develop measurement and testing methods for continuous and discrete traits based on the same foundational principles. Filling this gap will enable the testing of phylogenetic signals to incorporate more comprehensive trait information.

Phylogenetic signals have been widely used in the ecological and evolutionary research area, such as interspecific interactions (Nuismer and Harmon 2015), spatial distribution of species (Pashirzad et al. 2018), community assembly (Li et al. 2017), and community vulnerability and adaptation to climate change (Li et al. 2019; Melero et al. 2022). Some functions of organisms or communities are not determined by a single trait but by a combination of multiple traits. For example, drought resistance was found to be affected by total plant biomass, leaf mass ratio, and leaf area to root mass ratio together (Matías et al. 2012). Combinations of multiple traits have been widely used to explain community assembly (Barbour et al. 2015; Dwyer and Laughlin 2017) and ecosystem functions (De Bello et al. 2010; Laliberté and Legendre 2010). However, when a phylogenetic signal is used to explain these processes, the most commonly used indices can only individually detect phylogenetic signals for each trait (Münkemüller et al. 2012). Some studies have attempted to detect phylogenetic signals in multiple trait combinations. Zheng et al. (2009) incorporated observed data into a multiple trait Ornstein–Uhlenbeck (OU) model and regarded the statistical credibility of stabilizing selection within the model as a test for phylogenetic signals. However, their research did not develop a specialized measure for phylogenetic signals and was distinctly different from the widely used explicit methods for testing phylogenetic signals. Saito et al. (2016) proposed a more flexible distance‐based approach that measured phylogenetic signals using the Mantel test between phylogenetic and trait distances. Their method relied on Gower's distance to compute dissimilarity matrices and used the correlation coefficient as a proxy for phylogenetic signal strength. However, a statistically significant result from the Mantel test does not necessarily indicate the presence of phylogenetic signal. It is only when the correlation coefficient is positive that the presence of phylogenetic signal can be confirmed. This approach can be misused, especially when the correlation coefficient is negative. What is more, this approach does not detect phylogenetic signals by comparing the distances from phylogenies and traits, and thus is not in accordance with the definition provided by Blomberg and Garland Jr (2002). Therefore, it is necessary to develop more rigorous phylogenetic signal detection methods that can simultaneously assess multiple trait combinations.

Here, we developed a new method called the “*M* statistic” to detect the phylogenetic signals in both types of trait variables (continuous and discrete) as well as multiple trait combinations. This new method employed a distance‐based approach similar to that of Saito et al. (2016); however, the metrics constructed here followed a more rigorous definition of phylogenetic signals, diverging from reliance on correlation test results. The method's ability to handle various types of traits is derived from the use of Gower's distance in the calculation of the *M* statistic, which converts different types of traits into distances. This makes our method a versatile tool for phylogenetic signal detection. By applying the method to a simulated dataset, we compared the performance of the *M* statistic with that of the commonly used methods (Abouheif's *C*_mean_, Moran's *I*, Blomberg's *K*, and Pagel's *λ*) for continuous traits and with *D* and *δ* statistics for discrete traits in detecting phylogenetic signals across different sample sizes. The *M* statistic was also used to test the phylogenetic signals in multiple trait combinations extracted from the simulated dataset. We then demonstrated the utility of our new method using trait data of turtles (Testudines) and discussed opportunities for future applications of the method. An R package “phylosignalDB” was developed to facilitate all computations (see Date availability statement).

## 2 Materials and Methods

### 2.1 *M* Statistic

The novel approach proposed in this study strictly adheres to the definition of phylogenetic signals, formulates an index, and develops a testing method in strict accordance with the definition instead of relying on correlation analysis or evolutionary models. Blomberg and Garland Jr (2002) provided a widely accepted definition of the phylogenetic signal, which is the “tendency for related species to resemble each other more than they resemble species drawn at random from the tree”. The phrase “resemble each other” within the definition encapsulates the concept of species exhibiting trait values that are similar to one another. The degree of similarity can be quantified using similarity or dissimilarity indices (often expressed as 1 − similarity). These two coupled indices are commonly employed in ecological studies to quantify the comparative divergence or convergence of traits across communities and among various species (De Bello et al. 2021). The phrase “related species” emphasizes the closeness of phylogenetic relationships among species, which is commonly measured by the phylogenetic pairwise distance among species (also called phylogenetic dissimilarity if the distance is scaled between 0 and 1; Tucker et al. 2017). Considering that dissimilarity is a standardized form of distance, and the two terms are largely synonymous in most contexts, with “distance” being more commonly used, this article will use the term “distance” to refer to the differences between species in certain traits.

Phylogenetic signals encompass two sources of pairwise distances among species, derived separately from trait data and phylogeny. Calculating distances using trait data is more complex, mainly because the statistical or variable types of trait data are diverse and may be nominal, ordinal, interval, or a combination of these types (Pavoine et al. 2009). Trait distances were uniformly calculated using Gower's distance (Gower 1971), which has been demonstrated to be a suitable method for distance calculations using mixed‐type data in ecology (Pavoine et al. 2009; Saito et al. 2016). Gower's distance can handle both quantitative and qualitative traits, making it a versatile tool for phylogenetic signal detection. For quantitative traits, Gower's distance standardizes the differences by the maximum possible difference in the dataset, ensuring that all distances are scaled between 0 and 1. For qualitative traits, Gower's distance uses a binary approach to calculate the presence or absence of a trait. Gower's distance can be calculated using the gowdis function in the FD package (Laliberté and Legendre 2010), daisy function in the cluster package (Kaufman and Rousseeuw 2009), and dist.ktab function in the ade4 package (Pavoine et al. 2009; Dray and Dufour 2007). De Bello et al. (2021) provide the gowdis and trova functions, which not only handle general quantitative and categorical variables but also manage more complex variable types, such as fuzzy‐coded dummy variables and multiple variables representing a single characteristic.

We equivalently expressed the textual definition of the phylogenetic signal as an inequality equation of the two distances and constructed the *M* statistic. We used *d* ^phylo^ to represent phylogenetic distance and *d* ^trait^ to represent trait distance, and *d* ^trait^ was calculated using Gower's distance, which allows our method to handle any type of trait. Considering three species *i*, *j*, and *k*, if $d_{\mathrm{ij}}^{\text{phylo}}<d_{\mathrm{ik}}^{\text{phylo}}$ then, the “related species” *i* and *j* are more closely related on the phylogenetic tree than are the species *i* and *k*. According to the definition, if there is a significant phylogenetic signal, the trait values of species *i* and *j* are more likely to “resemble each other” than those between species *i* and *k*. It is expressed as an inequality as follows:

(1) $$d_{\mathrm{ij}}^{\text{phylo}}<d_{\mathrm{ik}}^{\text{phylo}}\Rightarrow d_{\mathrm{ij}}^{\text{trait}}<d_{\mathrm{ik}}^{\text{trait}}$$

If we consider it as mapping from phylogeny to traits, this inequality equation is referred to as a monotonicity constraint in multidimensional scaling theory (Cox and Cox 2000). The overall degree to which phylogeny‐to‐trait data mapping conforms to the monotonicity constraint for all species serves as a measure of the strength of the phylogenetic signal, called the *M* statistic. The *M* statistic ranges from 0 to 1. Theoretically, the calculation of the *M* statistic can be performed by iterating through all possible combinations of the three species (*i*, *j*, *k*) and determining whether they satisfy the monotonicity constraint. The proportion of combinations that satisfy the monotonicity constraint of all possible combinations is referred to as the *M* value. However, this is not a good choice, because the number of combinations to be iterated can be very large. Considering that random permutation simulations need to be performed (usually at least 999 times), this leads to excessive consumption of computational resources and prolonged computation times.

**FIGURE 1:**
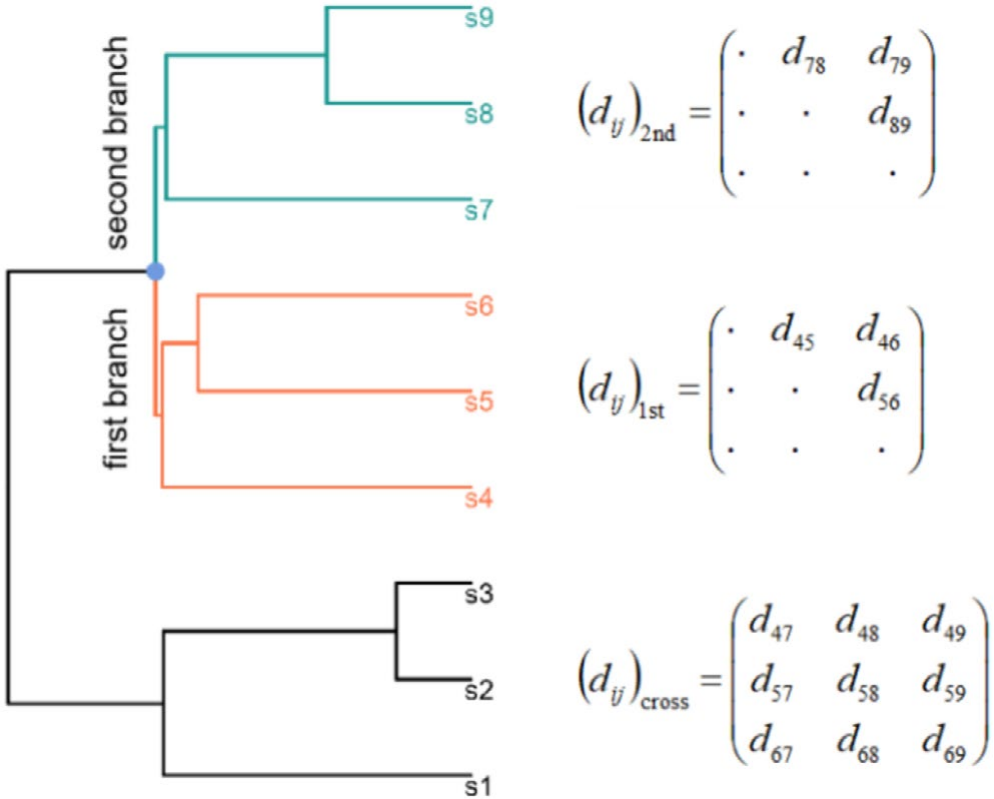
Schematic diagram of the *M* statistic. Each tip represents a species, and *d* denotes the distance.

This study proposed the “subtree method” as an alternative to the brute force method, effectively utilizing the phylogenetic topological relationships between species to reduce computational load. Internal nodes with at least three tips as descendants were considered the roots of the subtrees, and the phylogenetic tree was decomposed into a series of subtrees. A phylogenetic tree must be dichotomous, meaning that each internal node has exactly two child nodes. First, we determined whether the two branches emanating from the root of the subtree satisfy a monotonicity constraint (Figure 1). The two branches originally did not have a sequence of precedence; however, for convenience in differentiation, they were referred to as the first and second branches. Within each branch, the average pairwise distance among the tips, calculated as the average of all pairwise distances among the first or second branch of the phylogenetic tree was denoted as ${\bar{d}}_{1\mathrm{st}}$ for the first branch and ${\bar{d}}_{2\mathrm{nd}}$ for the second branch. For single‐tip branches, we set the distance to 0, indicating that the distance between an entity and itself is zero. The average interwoven mixed distance between the branches is denoted as ${\bar{d}}_{\text{cross}}$, calculated as the average of all pairwise distances between the tips in the first branch and those in the second branch. Phylogenetic distances inherently satisfy ${\bar{d}}_{1\mathrm{st}}^{\text{phylo}}$ < ${\bar{d}}_{\text{cross}}^{\text{phylo}}$ and ${\bar{d}}_{2\mathrm{nd}}^{\text{phylo}}$ < ${\bar{d}}_{\text{cross}}^{\text{phylo}}$. If the monotonicity constraint of Equation (1) is satisfied, then the two inequalities for trait distances (${\bar{d}}_{1\mathrm{st}}^{\text{trait}}$ ≤ ${\bar{d}}_{\text{cross}}^{\text{trait}}$ and ${\bar{d}}_{2\mathrm{nd}}^{\text{trait}}$ ≤ ${\bar{d}}_{\text{cross}}^{\text{trait}}$) should also very likely hold true. By assigning equal weights to the two branches, the degree to which the monotonicity constraint is satisfied for the *i*‐th subtree can be represented by score *m*_*i*_:

(2) $$m_{i}={\frac{1}{2}}^{*}\left\{\begin{array}{ll} 1 & \text{if}\;{\bar{d}}_{1\mathrm{st}}^{\text{trait}}\leq {\bar{d}}_{\text{cross}}^{\text{trait}}\\ 0 & \text{if}\;{\bar{d}}_{1\mathrm{st}}^{\text{trait}}>{\bar{d}}_{\text{cross}}^{\text{trait}} \end{array}\right.+{\frac{1}{2}}^{*}\left\{\begin{array}{ll} 1 & \text{if}\;{\bar{d}}_{2\mathrm{nd}}^{\text{trait}}\leq {\bar{d}}_{\text{cross}}^{\text{trait}}\\ 0 & \text{if}\;{\bar{d}}_{2\mathrm{nd}}^{\text{trait}}>{\bar{d}}_{\text{cross}}^{\text{trait}} \end{array}\right.$$

By a ggregating the scores of all subtrees (with the total number of subtrees denoted as *n*) and calculating the mean, we can obtain an overall score for the degree to which all species conform to the monotonicity constraint, which is a value between 0 and 1. This score is the *M* statistic, calculated as:

(3) $$M=\frac{1}{n}\sum_{i=1}^{n}m_{i}$$

The *M* statistic is tested using a random permutation test, a method also employed for Abouheif's *C*_mean_, Moran's *I*, and Blomberg's *K* indices (Münkemüller et al. 2012). It also aligns with the phylogenetic signal definition that implies “species drawn at random from the tree.” The larger the *M* value, the greater is the degree of satisfaction with the monotonicity constraint, indicating that the closer is the phylogenetic distance between species, the closer are their trait distances. The observed *M* value is considered significant if it exceeds most of the null *M* values obtained from random permutations, suggesting the rejection of the null hypothesis. This significance is determined using a non‐parametric *p* value based on the rank of the observed *M* within the null distribution. For a right‐tailed test, the observed *M* value is considered significant at the 5% level if it ranks below the 5th percentile, and at the 1% level if it ranks below the 1st percentile.

### 2.2 Design of a Simulation Study

A simulation study was designed to validate the phylogenetic signal detection capability of the *M* statistic. Phylogenetic trees and continuous traits were simulated using the simulation methods described by Münkemüller et al. (2012), allowing for comparison with results from other studies.

#### 2.2.1 Phylogeny

Phylogenetic trees were randomly generated using the sim.bdtree function from the geiger package (Pennell et al. 2014) with a pure‐birth setting (birth rate, 0.05; death rate, 0). The number of species (tips) in these phylogenetic trees was designed with gradients of 20, 50, 100, 250, and 500, covering almost all application scenarios. The simulated phylogenetic trees were ultrametric and dichotomous.

#### 2.2.2 Continuous Traits

A priori indicators of phylogenetic signal content (intensity) were required for the simulated traits to assess the testing capability of the phylogenetic methods for these differentiated traits. Continuous traits were derived from the weighted aggregation of trait_BM_, which followed Brownian motion (BM) along the phylogenetic tree, and trait_rand_, which was derived from the random shuffling of trait values:

(4) $${\text{trait}}_{\mathrm{con}}=w^{*}{\text{trait}}_{\mathrm{BM}}+\left(1-w\right)*{\text{trait}}_{\mathrm{rand}}$$

Weight coefficients (*w*) were assigned values in increments of 0.1 between 0 and 1. The variable trait_BM_ was generated using the rTraitCont function from the ape package (Paradis and Schliep 2019) with a root value of 0 and a standard deviation of 0.01. The variable trait_rand_ was obtained by randomly permuting the trait_BM_ values. The intensity coefficient (*w*) can be considered the true value of the phylogenetic signal content (intensity) for the simulated traits. When *w* = 1, the phylogenetic signal was unequivocal. Conversely, *w* = 0 indicated that the trait values consisted solely of random noise and contained no phylogenetic signals. The *M* statistic was applied alongside traditional methods (Abouheif's *C*_mean_, Moran's *I*, Blomberg's *K*, and Pagel's *λ*) to test the phylogenetic signals of the continuous traits. This would test how the results of the new method respond to different intensities of Brownian motion in trait evolution and compare its performance with those of traditional approaches. The measurements and tests of these traditional methods were conducted using the phyloSignal function in the phylosignal package (Keck et al. 2016).

#### 2.2.3 Discrete Traits

Discrete traits with varying intensities of phylogenetic signals were obtained using a two‐step process. First, we simulated the evolution of a discrete character along a phylogeny using a Markov (MK) model to obtain discrete traits trait_MK_ with clear a priori phylogenetic signals. Then a subset of species (the proportion of the subset is denoted by *w*) was extracted from the tips of the phylogenetic tree, with their trait values remaining unchanged. The trait values of the remaining species (with proportion (1 − *w*)) were randomly permuted (shuffled). The discrete traits ultimately obtained can be considered to have retained the Markov intensity at a proportion of *w* during the process of evolution. Similar to the simulation of continuous traits, intensity coefficients (*w*) were assigned values in increments of 0.1 between 0 and 1. Discrete traits were generated using the rTraitDisc function from the ape package with an equal change rate of 0.01 and a series of designated number of states. The number of states for discrete traits was designed with a gradient of 2, 3, 5, and 10 to explore the sensitivity of *M* to the number of trait states. The *M* statistic, *D* statistic, and *δ* statistic were applied to the simulated discrete traits to test for phylogenetic signals. The *M* statistic was applied to simulated traits with the number of states being 2, 3, 5, and 10. The *D* statistic was applied to simulated traits with the number of states being 2, calculated using the phylo.d() function from the caper package (Fritz and Purvis 2010; Orme et al. 2023). Although the *δ* statistic is theoretically not limited by the number of states of discrete traits, the available function script (Borges et al. 2019) runs slowly, so we only calculated it for simulated traits with two states and set the number of random permutations to 199. For the *M* statistic and *D* statistic, the number of random permutations was set to 999.

#### 2.2.4 Trait Combinations

The phylogenetic signal detection capability of *M* in handling multiple traits was assessed. Constrained by computational capacity, the free combination of a large number of traits can generate a heavy computational workload, which hinders the execution of the simulation. Here, the simulation involved only a combination of two traits that were directly copied from the previously simulated individual continuous and discrete traits. Specifically, a combination of continuous traits consisted of two independent continuous traits that evolved with the Brownian motion intensities of *w*_1_ and *w*_2_. Both *w*_1_ and *w*_2_ were assigned values in increments of 0.1 between 0 and 1. The number of species was set at two levels (50 and 250), resulting in 242 trait combinations. The construction process of discrete trait combinations was similar to that of continuous traits, with intensity coefficients *w*_1_ and *w*_2_ representing the strength of the Markov model in the trait evolution process. The number of states for discrete traits was designed with a gradient of 2, 3, 5, and 10, consistent with the individual discrete traits. Species size was set to 250 for illustrative purposes, yielding 484 discrete trait combinations. *M* was used to measure and test the phylogenetic signals of these trait combinations and to obtain the response of the test results to the intensity coefficients *w*_1_ and *w*_2_.

All simulations were repeated 100 times. When conducting tests of the null model, 999 random permutations (shuffles) were performed in both the *M* statistic and traditional indices (Abouheif's *C*_mean_, Moran's *I*, Blomberg's *K*, and Pagel's *λ*).

### 2.3 Experimental Case Study

In the present study, we prepared an ecological trait dataset for turtles and used the *M* statistic to detect phylogenetic signals. The dataset was derived from the recently published ReptTraits dataset (Oskyrko et al. 2024), extracting species classified under the major Testudines group (comprising 361 species). Only those ecological traits having trait records for more than 50% of the species were retained. We used the maximum clade credibility tree with 288 tips provided by Thomson et al. (2021) for the phylogenetic analysis of turtles. Only species that were present in both the ReptTraits dataset and the turtle phylogenetic tree were selected for this study. Ultimately, our turtle trait dataset comprised 240 species, encompassing 5 morphological traits, 2 behavioral traits, 2 life history traits, 5 habitat variables, and 2 variables related to species conservation status. For further details, please refer to Table 1 and Appendix S1.

**TABLE 1:** Traits of turtles (Testudines) selected in this study and the results of phylogenetic signal detection using the *M* statistic.

Trait group	Ecological trait	*M*	*p*	Statistical type	Variable type	Species number^a^
Morphological traits	Maximum longevity (years)	0.659	**0.002**	Quantitative	Continuous	183
	Maximum body mass (g)	0.639	**0.001**	Quantitative	Continuous	238
	Maximum length (“SVL,” mm)/straight carapace length for turtles (“SCL,” mm)	0.620	0.102	Quantitative	Continuous	137
	Dorsal color	0.727	**0.001**	Nominal	Discrete	191
	Dorsal pattern	0.683	**0.002**	Nominal	Discrete	186
	Combination	0.685	**0.001**	Multivariate	Multiple	111
Behavioral traits	Diet	0.717	**0.001**	Nominal	Discrete	124
	Active time	0.644	0.111	Nominal	Discrete	143
	Combination/group	0.695	**0.016**	Multivariate	Multiple	97
Life history traits	Hatchling/neonate mass (g)	0.740	**0.001**	Quantitative	Continuous	146
	Mean number of offspring per litter or number of eggs per clutch	0.678	**0.001**	Quantitative	Continuous	204
	Combination/group	0.735	**0.001**	Multivariate	Multiple	146
Habitat variables	Main biogeographic region	0.918	**0.001**	Nominal	Discrete	240
	Microhabitat	0.677	**0.004**	Nominal	Discrete	234
	Habitat type	0.741	**0.001**	Nominal	Discrete	216
	Mean annual temperature (°C)	0.706	**0.001**	Quantitative	Continuous	151
	Temperature seasonality (standard deviation × 100)	0.691	**0.001**	Quantitative	Continuous	151
	Combination/group	0.753	**0.001**	Multivariate	Multiple	146
Conservation status	IUCN redlist assessment	0.672	**0.001**	Ordinal	Discrete	190
	IUCN population trend	0.625	**0.020**	Ordinal	Discrete	188
	Combination/group	0.625	**0.012**	Multivariate	Multiple	188

*Note:* Bolded *p* values indicate a significant phylogenetic signal (*p* ≤ 0.05).

^a^ For a single trait, the species number refers to the number of species that possess this trait without any missing values. For a combination of traits, the species number refers to the number of species that simultaneously possess all these individual traits. Data from Oskyrko et al. (2024).

The *M* statistic was used to measure and test the phylogenetic signals for these traits. First, we assessed whether each individual trait exhibited phylogenetic signals; subsequently, these traits were paired to form combinations, and the distribution of these combinations was tested for phylogenetic signals.

### 2.4 R Package

The *M* statistic can be measured and tested using the R (R Core Team 2024) package “phylosignalDB Version: 0.2.2” (exploring phylogenetic signals using Distance‐Based methods). The R package is available at CRAN: https://cran.r‐project.org/web/packages/phylosignalDB/.

## 3 Results

### 3.1 Performance

#### 3.1.1 Testing Phylogenetic Signals in Continuous Traits

**FIGURE 2:**
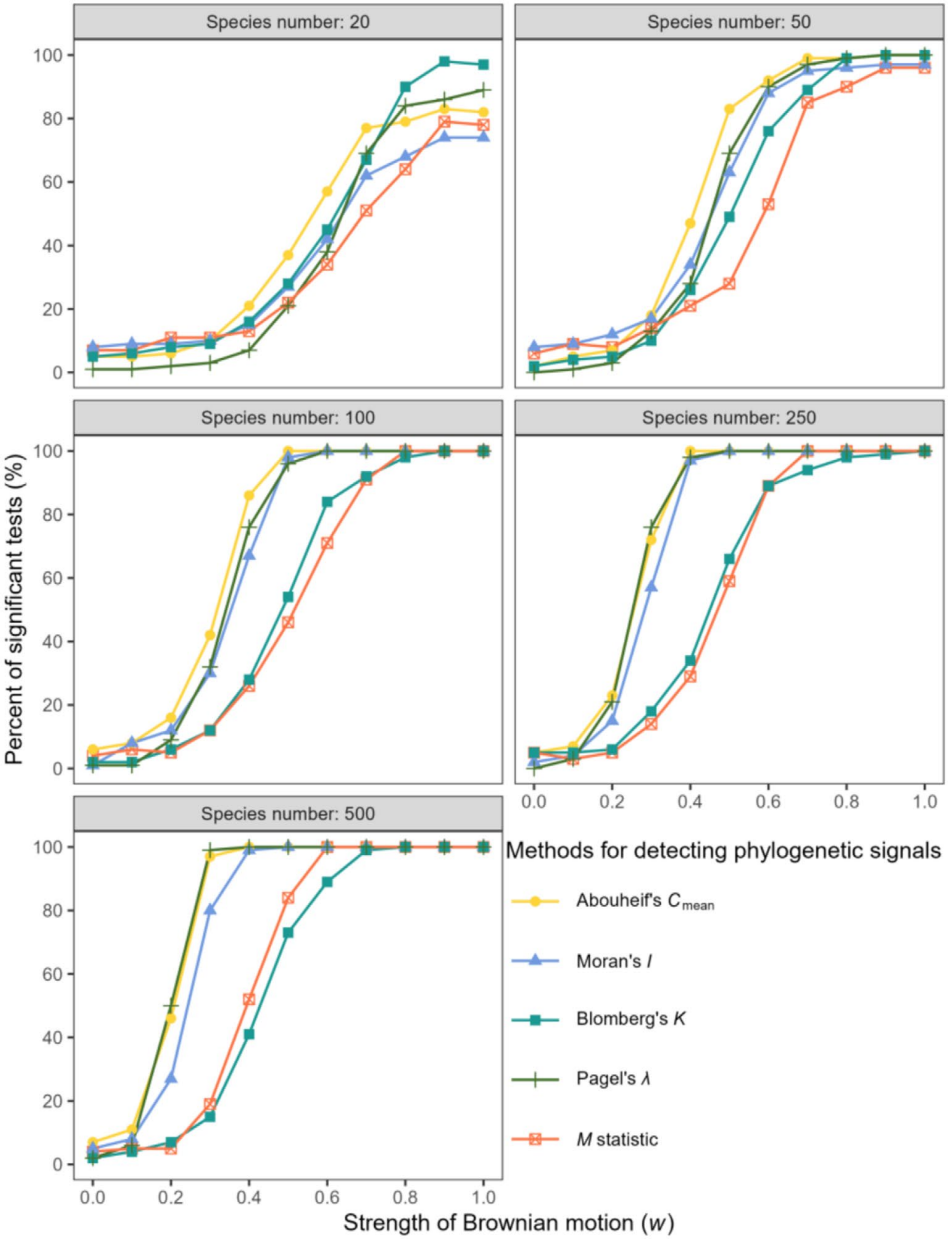
Response of five phylogenetic signal test methods to the increasing strength of Brownian motion for different species numbers. The rejection rate of the null hypothesis (the detection rate of phylogenetic signals) is shown.

The performance of our new method was tested using simulated continuous trait data and compared with that of four commonly used methods (Figure 2). The detection rate of phylogenetic signals by the *M* statistic increased with the strength of the Brownian motion and responded to the species number (sample size). The detection rate of phylogenetic signals remained low when the strength of Brownian motion was below 0.2, but it then increased with increasing departure from random trait evolution, and phylogenetic signals were identified with moderate Brownian motion. The maximum achievable detection rate and its occurrence times varied with the species number. The maximum achievable detection rate reached 100% only when the sizes of the phylogenies were sufficiently large (species number ≥ 100). For example, the detection rate reached a maximum value (100%) when the strength of Brownian motion reached 0.7 at a sample size of 500. It reached a maximum value (approximately 90%) when the strength of Brownian motion reached 0.9 at a sample size of 50.

*M* performed as well as the existing widely used Abouheif's *C*_mean_, Moran's *I*, Blomberg's *K*, and Pagel's *λ*. In general, *M* performed more similarly to Blomberg's *K*, and both were less sensitive, reaching their maximum values under stronger Brownian motion compared to the other three indices (Figure 2).

#### 3.1.2 Testing Phylogenetic Signals in Discrete Traits

**FIGURE 3:**
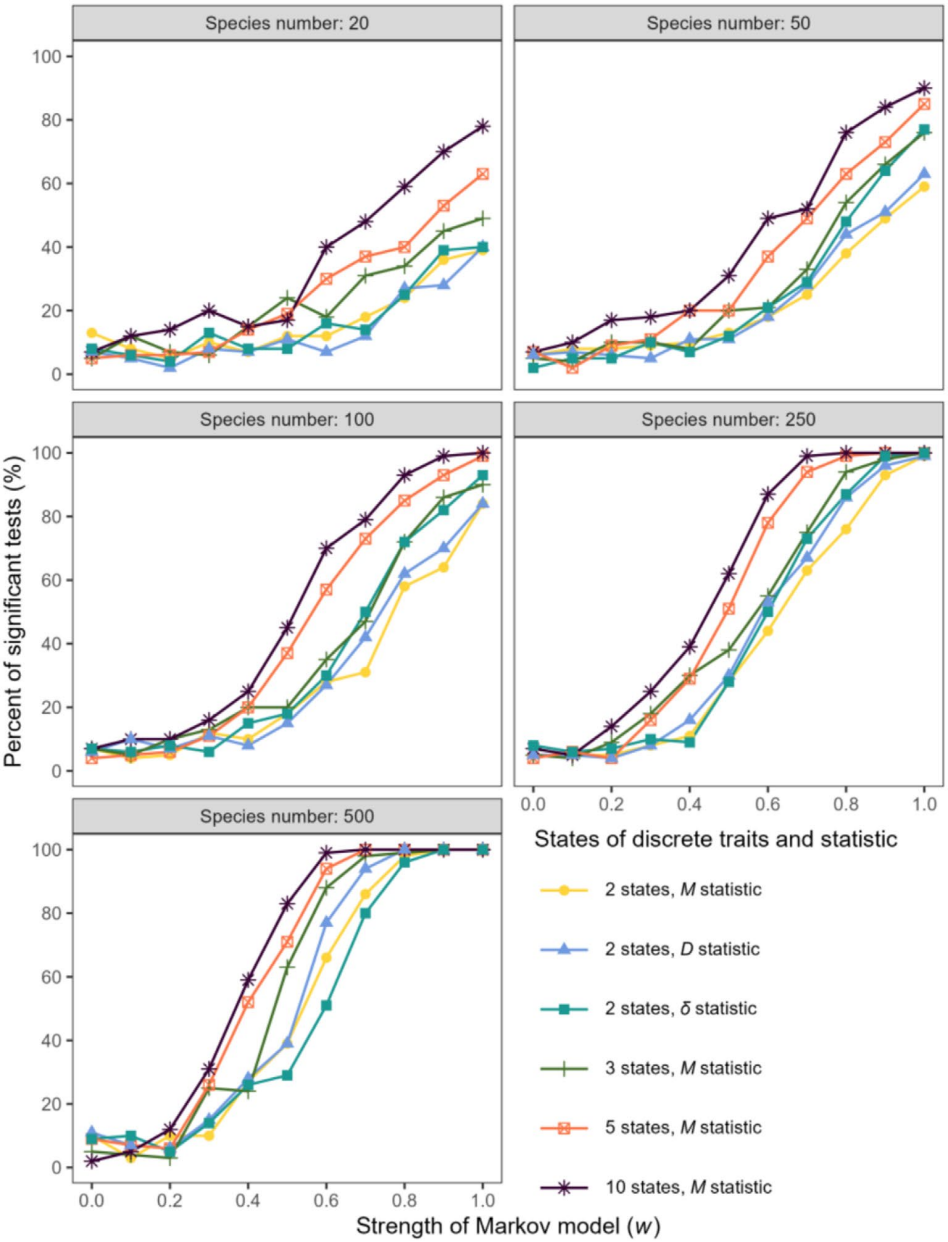
Response of phylogenetic signal tests of discrete variables (state number of discrete traits = 2, 3, 5, or 10) by the *M* statistic, and of discrete traits = 2 by the *D* and *δ* statistics to the increasing strength of the Markov model for different species numbers. The rejection rate of the null hypothesis (the detection rate of phylogenetic signals) is shown.

The response of the phylogenetic signal test to different strengths of the Markov model was examined with state numbers of 2, 3, 5, and 10 to test the performance of *M* in dealing with discrete variables (Figure 3). The sensitivity of the test to the strength of the Markov model increased with the size of the phylogenies and the state number of discrete traits. When the species number was small, the performance of *M* in handling discrete variables was inferior to that in handling continuous variables (Figures 2 and 3). When the species number was ≤ 50, the detection rate of phylogenetic signals increased slowly with the strength of the Markov model, and phylogenetic signals were not identified with moderate Markov model intensity. When the species number exceeded 250, the performance of *M* in handling discrete variables was almost as good as that when processing continuous variables. The phylogenetic signals were identified with a moderate strength of the Markov model when the species number was 250 or 500, and reached the maximum value (100%) earlier than those for lower species numbers. We discovered a pattern wherein the larger the phylogenetic size, the earlier the maximum value emerged. Additionally, the sensitivity of the test to the strength of the Markov model was the strongest when the number of states of the discrete trait was 10.

The performance of the *M* statistic was very similar to that of the *D* and *δ* statistics when the state number of discrete traits was 2. Their detection rate of phylogenetic signals increased with the increasing strength of the Markov model and also improved with the increasing number of species (Figure 3).

#### 3.1.3 Testing Phylogenetic Signals in Combinations of Two Traits

**FIGURE 4:**
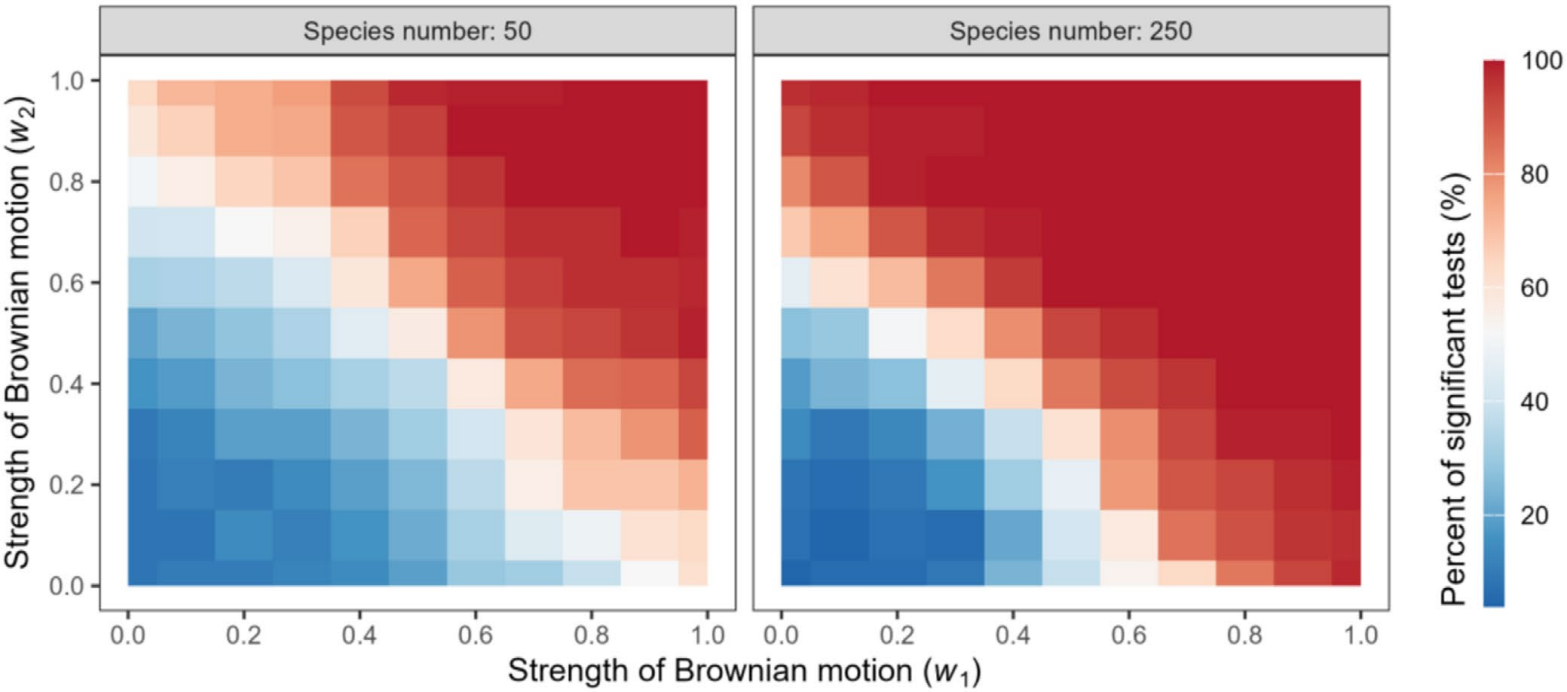
Response of phylogenetic signal tests by the *M* statistic to the increasing strength of Brownian motion for two continuous traits. Different colors represent the percentage range of significant tests.

**FIGURE 5:**
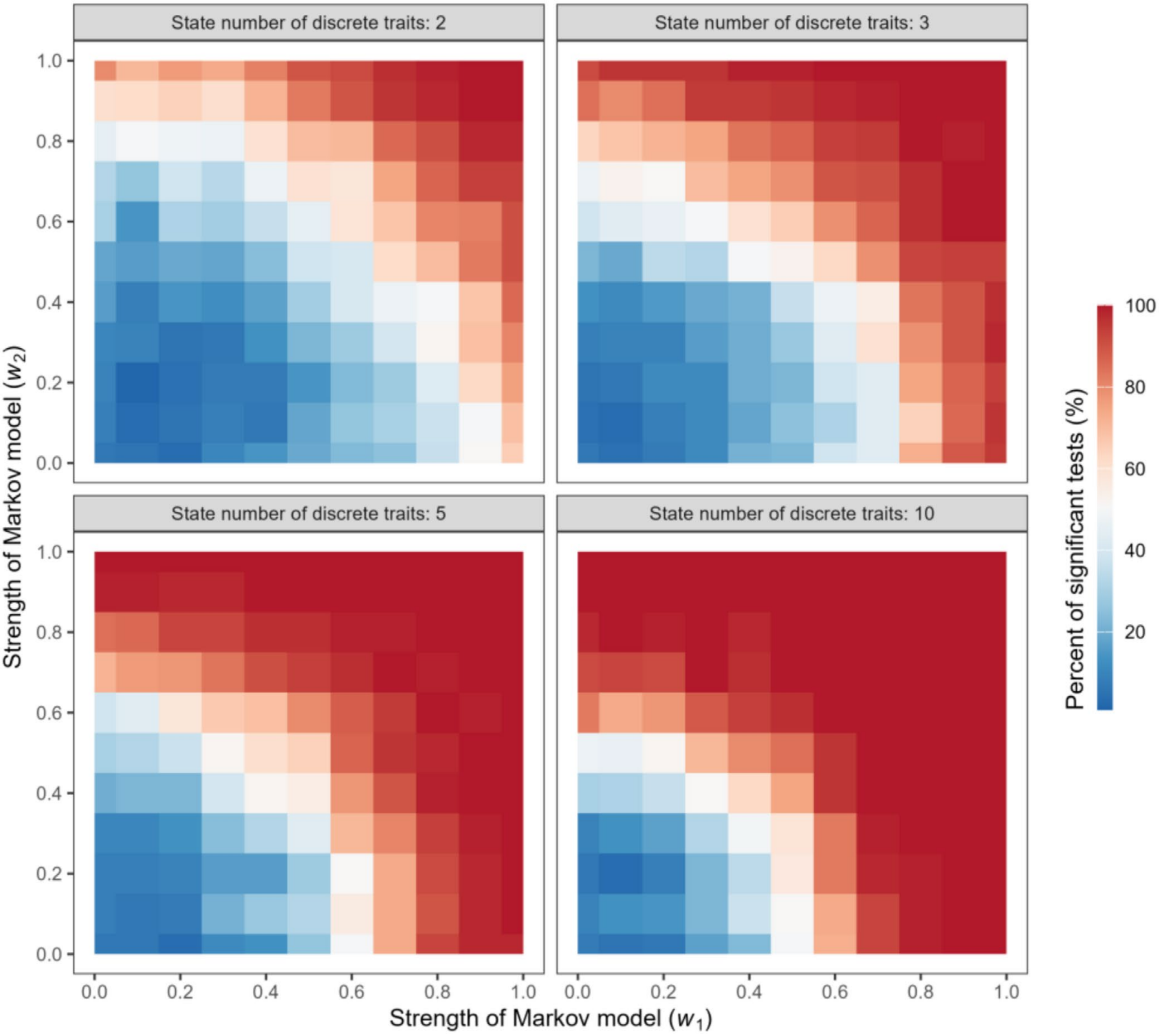
Response of phylogenetic signal tests by the *M* statistic to the increasing strength of the Markov model for two discrete traits with a species number of 250. Different colors represent the percentage range of significant tests.

Owing to the limitations in computational capacity, we used the processing of two traits as an example to illustrate the capability of the *M* statistic to test phylogenetic signals in multiple trait combinations. The detection rate of phylogenetic signals increased with an increase in the strength of the Brownian motion or Markov model of each single variable separately (along the horizontal or vertical axes) and increased when the strength of the Brownian motion or Markov model of both variables increased together (along the diagonal; Figures 4 and 5). The statistical power improved when two traits were tested together. The detection rate of phylogenetic signals reached 100% earlier when the two traits were tested together than when they were tested separately. For continuous variables, the test of phylogenetic signal responded to the sample size. At the same strength of Brownian motion, the detection rate of phylogenetic signals for a sample size of 250 was higher than that for a sample size of 50 (Figure 4). For discrete traits, when the sample size was 250, the detection rate of phylogenetic signals was higher for a larger number of discrete variable states (Figure 5).

### 3.2 An Application: Measuring Phylogenetic Signals in Turtle Traits

According to the results of the *M* statistic, most of the selected ecological traits of turtles showed significant phylogenetic signals (*p* < 0.05), except for “maximum length” and “active time” (Table 1). The phylogenetic signal values ranged from 0.625 to 0.918. In addition to the individual traits, the combination of traits in each group exhibited significant phylogenetic signals (Table 1). The phylogenetic signals of each combination were between the maximum and minimum phylogenetic signals of the constituent individual traits.

## 4 Discussion

We developed a novel phylogenetic method that can be applied to a wide range of biological data, including continuous, discrete, and combinations of trait variables. Employing a uniform analytical approach to handle various traits can prevent the lack of comparability of results that may arise from using different methods to separately handle continuous and discrete traits. This capability is largely attributed to our reliance on Gower's distance, which enables the method to handle any type of trait. Furthermore, examining a set of traits that collectively explain a particular behavior or function as a composite for phylogenetic signal detection can enhance the association between phylogenetics and ecological processes.

### 4.1 Performance and Influencing Factors of the *M* Statistic

In comparison to the other four methods, the results demonstrated that *M* performed commendably and exhibited a close affinity for Blomberg's *K*. Both methods exhibited relatively low sensitivity to the strength of Brownian motion for individual continuous variables (Figure 2). This result is similar to that of Münkemüller et al. (2012), who also found that Blomberg's *K* had a low sensitivity to the true underlying patterns of phylogenetic signals. In certain contexts, the low sensitivities of *M* and Blomberg's *K* may not necessarily be disadvantageous. These can be seen as conservative approaches for detecting phylogenetic signals, potentially reducing the risk of false positives (type I error). This may be particularly valuable in cases where the true phylogenetic signal is subtle or the data are noisy, thereby avoiding overinterpretation of the results. Blomberg's *K* has been revealed to be highly sensitive to inaccuracies in branch‐length information, leading to type I errors (Molina‐Venegas and Rodríguez 2017). In contrast, the *M* statistic is relatively insensitive to such inaccuracies because it focuses mainly on the topological structure of the phylogeny rather than on specific branch‐length information. When the phylogeny has a credible topological structure but suboptimal branch‐length information, the *M* statistic becomes a viable choice.

When the sample size was sufficiently large, *M* performed well for both continuous and discrete traits. Previous studies also support our finding that the statistical power of phylogenetic methods increases with sample size (Blomberg et al. 2003; Kamilar and Cooper 2013). Large sample sizes can provide sufficient data points to enhance the statistical power of the analysis, offer rich genetic diversity and variability, and reduce noise. In addition, the phylogenetic determination of discrete variables requires a larger sample size than that of continuous variables to detect significant phylogenetic signals (Figures 2 and 3). Phylogenetic signals in discrete traits are less likely to be detected (rejecting the null hypothesis) compared to those in continuous traits, primarily for the following reasons: First, compared to continuous variables, discrete variables typically have a limited range of possible values, which may result in low data granularity. Therefore, the observed sample differences may not be sufficiently significant to reflect overall population differences. Secondly, in the context of continuous traits, the values within a distance matrix are continuously distributed. In contrast, the distances calculated from the discrete traits are distributed as a finite set of discrete values. When the number of states is small, the number of possible distance values is limited, which can result in a low statistical power for the test. For example, when the trait is binary, the distance values are limited to two, which restricts the ability to distinguish the *M* values observed in the data from those obtained through random permutations. This makes it less likely to reject the null hypothesis (i.e., no signal). When the number of states for a discrete trait increases, the statistical power tends to increase, as observed in our simulation results (Figures 3 and 5). In extreme cases, when the number of states increases to infinity, the discrete trait approaches the characteristics of a continuous trait. A simulation analysis was conducted to examine the effect of the number of tips in phylogenetic trees on the signal detection capability of the *M* statistic (Figure S1). We suggest that the number of tips should be no less than 25 for continuous traits and at least 50 for discrete traits. Additionally, we have examined the performance of the *M* statistic when applied to SSE (State‐Dependent Speciation and Extinction) type models (Herrera‐Alsina et al. 2019). These models are particularly relevant for analyzing the interaction between traits (often discrete) and the phylogenetic tree. The *M* statistic can detect a phylogenetic signal in most of the simulated samples (Figure S2). However, when the crown age of the phylogenetic trees is shallow, leading to a small number of tips in the trees, it becomes difficult to detect the signal. Among the three models, the CR model is the easiest to detect signals, followed by the ETD model, and the CTD model is the least effective. It can be seen that the *M* statistic can be used to test for phylogenetic signals related to SSE type models.

After evaluating the performance of the *M* statistic using simulated trait data, we then demonstrated the utility of our new method using empirical trait data from turtles (Table 1). Some individual turtle traits were insufficient to detect significant phylogenetic signals; however, when groups of traits were combined, significant phylogenetic signals were detected (Table 1). In the phylogenetic signal testing conducted with simulated data, as the strength of the Brownian motion or the Markov model increased, trait combinations exhibited phylogenetic signals earlier than did individual traits (Figures 4 and 5). Zheng et al. (2009) also identified the failure of phylogenetic signal detection when dealing with single traits; however, when applied to a combination of traits, the statistical power was enhanced. Different traits may reflect various aspects of the evolutionary history of a species, and combining multiple traits can provide a more comprehensive set of evolutionary information, thereby reducing the bias or noise that may be present in individual traits.

### 4.2 Proposed Enhancements and Future Refinements

The *M* statistic is a promising tool for assessing phylogenetic signals; however, there are challenges that need to be addressed for continuous improvement. First, when calculating distances for multiple traits, the weighting of each trait can affect the results of the *M* statistic, adding complexity and uncertainty to the analysis of multiple trait phylogenetic signals; however, it may also enhance the potential to identify the importance of traits. Second, the *M* statistic has certain data quality requirements. Many missing values may diminish the statistical power of the *M* statistic, as is the case with other methods. Third, the behavior of the *M* statistic under various evolutionary models warrants an in‐depth study. Extensive simulations and empirical research are required to clarify the distribution characteristics of *M* values across different models to explore the potential of the *M* statistic in identifying patterns of trait evolution. Fourth, the interpretive power of the *M* statistic may depend on specific research objectives and contexts. However, its applicability and interpretive strength may vary for different biological systems or research questions and require further assessment. What's more, in practical research, phylogenies with polytomies are quite common due to the challenges in resolving all branching points. This is especially the case when constructing supertrees that integrate various smaller phylogenies and add missing species as polytomies guided by taxonomy (Davies et al. 2012; Molina‐Venegas and Rodríguez 2017). The current *M* statistic method, which is limited to dichotomous phylogenies, may somewhat restrict the potential applications of the *M* statistic. Therefore, future work will focus on refining the existing algorithm for the *M* statistic to make it suitable for phylogenies with polytomies, thereby broadening its utility in diverse research contexts. The efficiency of a statistical measure is crucial for its practical application, especially when dealing with large datasets or requiring extensive computations. Currently, the computation speed of our new package is slower than the phyloSignal() function in the phylosignal package (which includes Abouheif's *C*mean, Moran's *I*, Blomberg's *K*, and Pagel's *λ*) and the phylo.d() function in the caper package (*D* statistic), but faster than the *δ* statistic calculation script.

It is worth noting that using Gower's distance correctly is crucial, especially for complex trait variable types such as fuzzy‐coded dummy variables and multiple variables representing a single characteristic, to ensure accurate and reliable results. The methods for calculating Gower's distance for complex variable types are still under development. Some methods have been proposed but exist as hard‐to‐use code scripts without detailed documentation or examples (De Bello et al. 2010). In the future, we plan to develop more user‐friendly functions to calculate Gower's distance, which will be integrated into the phylosignalDB package.

## 5 Conclusion

We developed a novel approach capable of conducting phylogenetic signal detection in continuous and discrete traits, as well as in multiple trait combinations. The new method strictly adheres to the definition of the phylogenetic signals and detects them by comparing distances from phylogenies and traits. It has performed well in detecting phylogenetic signals by analyzing both simulated data and empirical trait data. It will enable a more comprehensive assessment of phylogenetic signals by incorporating a broader range of trait data.

## Author Contributions

**Liang Yao:** formal analysis (lead), funding acquisition (equal), methodology (equal), software (lead), visualization (lead), writing – review and editing (supporting). **Ye Yuan:** formal analysis (supporting), funding acquisition (equal), methodology (equal), software (supporting), visualization (supporting), writing – original draft (lead), writing – review and editing (lead).

## Conflicts of Interest

The authors declare no conflicts of interest.

## Supporting information


Appendix S1.

Appendix S2.

## Data Availability

The R package is available on CRAN (https://cran.r‐project.org/web/packages/phylosignalDB/) and at GitHub (https://github.com/anonymous‐eco/phylosignalDB). All data and code used to generate the figures and tables are provided in FigShare (https://doi.org/10.6084/m9.figshare.26315218.v1).
