# Supplementary material for: A Unified Method for Detecting Phylogenetic Signals in Continuous, Discrete, and Multiple Trait Combinations
Source: Ecol Evol. 2025 Mar 20;15(3):e71106. doi: 10.1002/ece3.71106 (PMC11925719; doi:10.1002/ece3.71106)
Supplement: Supplementary file 2 — Appendix S2. [file ECE3-15-e71106-s001.docx]

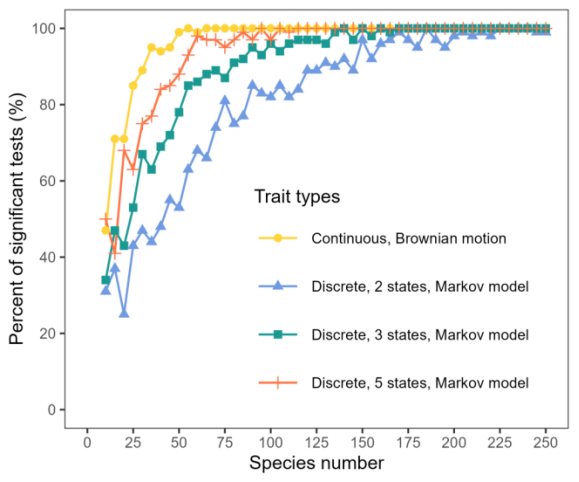


**Fig. S1**. A simulation analysis to examine the effect of the number of tips in phylogenetic trees on the signal detection capability of the *M* statistic. Phylogenetic trees were randomly generated using the sim.bdtree function from the geiger package with a pure-birth setting (birth rate, 0.05; death rate, 0). The continuous traits, which followed Brownian motion (BM) along the phylogenetic tree, was generated using the rTraitCont function from the ape package with a root value of 0 and standard deviation of 0.01. The number of species (tips) in these phylogenetic trees were designed from 10 to 250 stepped by 5. We simulated the evolution of a discrete character along a phylogeny using a Markov (MK) model to obtain discrete traits with clear a priori phylogenetic signals. The number of states for discrete traits was designed with a gradient of 2, 3, 5, and 10 to explore the sensitivity of *M* to the number of trait states. All simulations were repeated 100 times. When conducting tests of the null model, 999 random permutations (shuffles) were performed.


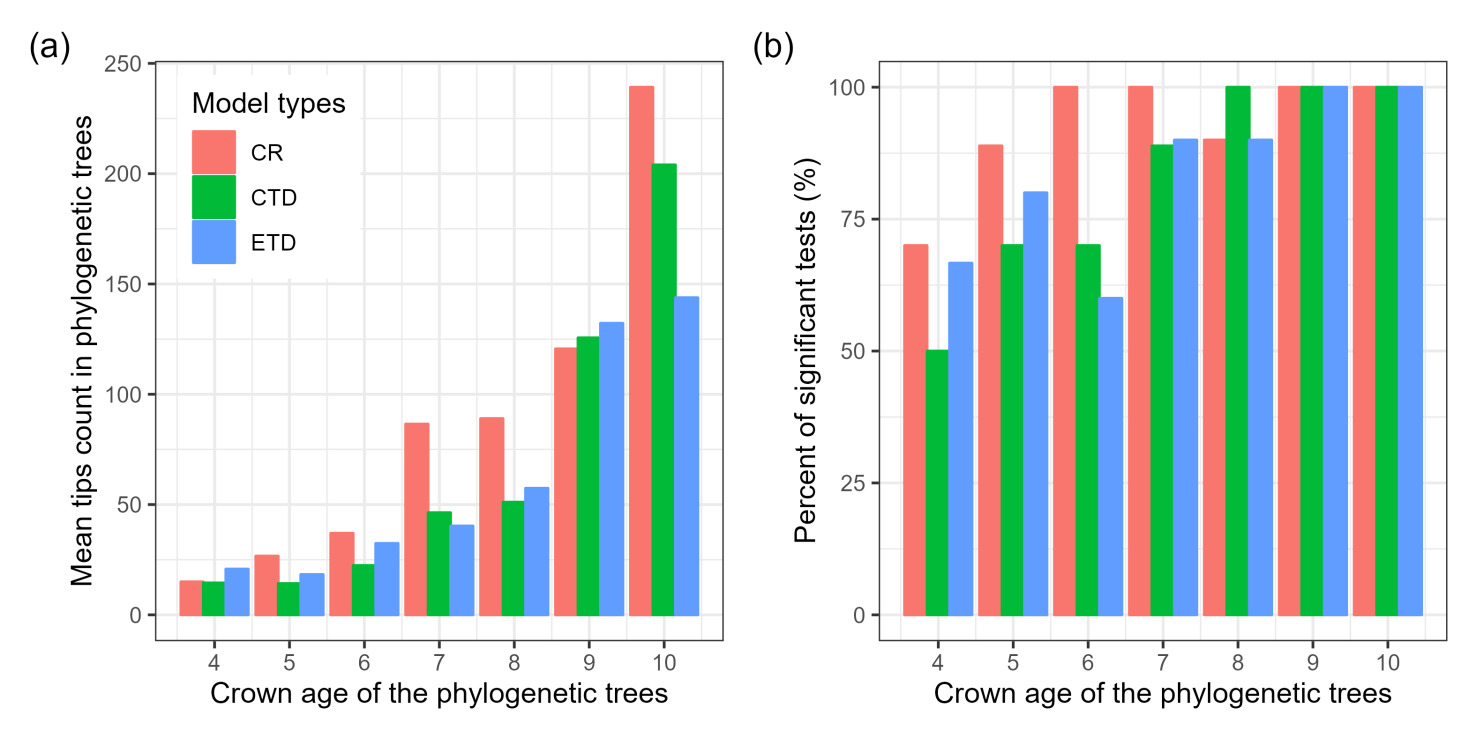


**Fig. S2**. Performance of the *M* statistic on SSE type models. The secSSE package was utilized to simulate phylogenetic trees and traits based on the SSE model. Only the case where the trait values are binary (state number = 2) was considered. The model of trait evolution was divided into CR (Constant Rate), CTD (Concealed Traits Diversification), and ETD (Examined Traits Diversification). The crown_age parameter of the phylogenetic tree was set from 4 to 10. We repeated the simulation 100 times, resulting in 100 * 3 * 7 phylogenetic trees and traits, and then used the *M* statistic to test for phylogenetic signals.
